# A SEMantic and EPisodic Memory Test (SEMEP) Developed within the Embodied Cognition Framework: Application to Normal Aging, Alzheimer's Disease and Semantic Dementia

**DOI:** 10.3389/fpsyg.2017.01493

**Published:** 2017-09-13

**Authors:** Guillaume T. Vallet, Carol Hudon, Nathalie Bier, Joël Macoir, Rémy Versace, Martine Simard

**Affiliations:** ^1^Centre de Recherche de l'IUGM, Université de Montréal Montreal, QC, Canada; ^2^Département de Psychologie, Université de Montréal Montreal, QC, Canada; ^3^Laboratoire de Psychologie Sociale et Cognitive, Centre National de la Recherche Scientifique, Université Clermont Auvergne Clermont-Ferrand, France; ^4^Département de Psychologie, Université Laval Quebec, QC, Canada; ^5^Centre de Recherche de l'Institut Universitaire en Santé Mentale de Québec Quebec, QC, Canada; ^6^Département de Réadaptation, Université Laval Quebec, QC, Canada; ^7^Laboratoire EMC, Université Lyon 2 Lyon, France

**Keywords:** long-term memory, embodied cognition, aging, Alzheimer's disease, semantic dementia

## Abstract

Embodiment has highlighted the importance of sensory-motor components in cognition. Perception and memory are thus very tightly bound together, and episodic and semantic memories should rely on the same grounded memory traces. Reduced perception should then directly reduce the ability to encode and retrieve an episodic memory, as in normal aging. Multimodal integration deficits, as in Alzheimer's disease, should lead to more severe episodic memory impairment. The present study introduces a new memory test developed to take into account these assumptions. The SEMEP (SEMantic-Episodic) memory test proposes to assess conjointly semantic and episodic knowledge across multiple tasks: semantic matching, naming, free recall, and recognition. The performance of young adults is compared to healthy elderly adults (HE), patients with Alzheimer's disease (AD), and patients with semantic dementia (SD). The results show specific patterns of performance between the groups. HE commit memory errors only for presented but not to be remembered items. AD patients present the worst episodic memory performance associated with intrusion errors (recall or recognition of items never presented). They were the only group to not benefit from a visual isolation (addition of a yellow background), a method known to increase the distinctiveness of the memory traces. Finally, SD patients suffer from the most severe semantic impairment. To conclude, confusion errors are common across all the elderly groups, whereas AD was the only group to exhibit regular intrusion errors and SD patients to show severe semantic impairment.

## Introduction

Embodiment has revolutionized how cognition is conceived (Glenberg et al., 2013) to highlight the role of sensory-motor components in cognitive processes (Vallet et al., 2016a). Applied to memory, it was shown repeatedly that semantic knowledge (i.e., knowledge about the world) might also be grounded in sensory-motor features (e.g., Vallet et al., 2010; Casasanto, 2011; Borghi, 2015), as episodic memory (i.e., personal and contextual memories, see Tulving, 1995). Semantic and episodic knowledge may thus share common memory traces, as stated by some memory models (e.g., Minverva II, Hintzman, 1990; Act-In, Versace et al., 2014). The present study proposes a memory test developed to take into account embodiment statements in young adults (YA), healthy elderly adults (HE), patients with Alzheimer's disease (AD) and patients with semantic dementia (SD). These populations exhibit variable levels of perceptual, episodic and semantic memory impairments that allow a differential approach assessing some of the core assumptions of embodiment.

Among the different kinds of memory models, multiple trace models (e.g., Hintzman, 1990) and embodied memory models (e.g., Versace et al., 2014) defines memory as an accumulation of episodic memory traces. All the traces are episodic in nature as all the characteristics of the ongoing event (sensory-motor, emotional, context…) are encoded. Therefore, the distinction between semantic and episodic memory is not in their nature, but rather how knowledge emerges from the activation of a selected (episodic knowledge), or reversely of multiple (semantic knowledge), traces based on the similarity between the target object and each of its traces. A direct consequence of the common traces hypothesis regards the neuropsychological evaluation. Instead of independently assessing semantic and episodic knowledge, the clinician might benefit from a parallel evaluation (see Greenberg and Verfaellie, 2010). This would mainly ensure that the concept to be learned (e.g., a list of words) is known by the patient (e.g., semantic relationship in a matching task), as well as accessible (e.g., lexical access through a naming task) to the patient.

Another consequence of an embodied approach to memory is to consider the role of perception into memory performance because memory traces remain grounded in their sensory components (Borghi, 2015; Brunel et al., 2015). It can be assumed that different levels of perceptual deficits should be associated with different kinds, or levels, of memory impairments. According to the Act-In memory model (Activation-Integration, Versace et al., 2014), the more distinctive a memory trace is, the more likely it can be retrieved (e.g., Brunel et al., 2013). Therefore, if knowledge is grounded in sensory components, a perceptual decline should directly be associated with lower encoding and retrieval performance. This is the case in normal aging in which the sensory and perceptual decline is significantly correlated with the cognitive decline (see Roberts and Allen, 2016, for a recent review). HE are also known to be more prone to memory errors than YA. These errors might be caused by executive (e.g., Meade et al., 2012) and perceptual deficits (e.g., Yeung et al., 2013). It could then be hypothesized that HE would commit more memory errors than YA, when they have to selectively learn one item among multiple presented items (source memory), and when the lures share common features with the target (especially for perceptual features, Butler et al., 2010).

Interestingly, these memory errors in aging have been associated with an hyper-binding of related or closely presented (in space or time) items (Campbell et al., 2014), which might be explained by their preserved, and perhaps enhanced, higher perceptual integration compared to YA (e.g., Laurienti et al., 2006). Therefore, HE should not falsely recall or recognize items that are not closely related to the targets, on the contrary to patients with AD. Indeed, AD is characterized by severe episodic memory deficits from encoding to recognition (Fleischman and Gabrieli, 1999). Yet, this population is also associated with a perceptual and sensory decline more severe than that in normal aging. Moreover, their higher perceptual functions, such as multimodal integration, are impaired (Delbeuck et al., 2007) which have been associated by some authors to their memory dysfunctions (e.g., Vallet et al., 2013, 2016b). The disconnection between the different parts of their brain can thus account for the impairment of multimodal integration and memory deficits (Delbeuck et al., 2003). More specifically, the disconnection between the hippocampus and adjacent or distant structures, such as parahippocampal and frontal regions (Rémy et al., 2015), is associated with an episodic memory deficit. These regions are involved in the retrieval of true memories (see Okado and Stark, 2003) which suggests that AD patients are more likely to commit errors for items never presented before, or related to the targets (see MacDuffie et al., 2012). However, classical memory approaches do not predict whether AD patients should commit or not more confusion errors than HE (Waldie and Kwong, 2003; Abe et al., 2011), on the contrary to embodiment in which confusion errors should be similar across these two groups since both groups show relatively similar low-level perceptual decline (see Vallet, 2015).

Furthermore, embodied memory models also assume the multimodal integration occurs during the retrieval of a memory trace, in addition of encoding (e.g., Zimmer et al., 2006), to dynamically bind the components of the trace (e.g., Brunel et al., 2013; Versace et al., 2014). Therefore, the addition of any multimodal components should negatively impair the memory performance of AD patients (Festa et al., 2005), even when this addition is known to increase the distinctiveness of the memory traces in other populations (for a review, see Schmidt, 1991). One can imagine that the disconnection syndrome will reduce the ability of AD patients to benefit from some perceptual isolation techniques such as adding a colored background. Isolation is a method commonly used to enhance the distinctiveness of a small set of items to be learned by giving them a particularity not shared with the other items, either from an intrinsic characteristic of the stimuli (e.g., Brunel et al., 2010), or from contextual manipulation (Oker et al., 2009).

The present article proposes a SEMantic EPisodic memory test (SEMEP) developed from an embodied cognition perspective adapted from the Pyramid and Palm Tree Test (PPTT, Howard and Patterson, 1992). Our goal is not to prove the different assumptions stated by the embodied cognition theories, but rather to illustrate how they could be applied within the clinical context of memory evaluation for differential diagnosis. The main assumptions taken into account are (1) the common memory traces for semantic and episodic knowledge, (2) the sensory-motor nature of the memory traces, and (3) the central role of integration in the emergence of episodic knowledge. It is expected that (1) performances in semantic tasks should directly impact performances in episodic tasks, (2) reduced perceptual ability should decrease episodic memory retrieval and (3) impaired multimodal integration should impair recall and recognition.

The first hypothesis is tested by using the same material in semantic tasks (matching and naming) and in episodic tasks (free recall and recognition), and by including patients with semantic dementia (SD). SD is a rare neurodegenerative disorder characterized by semantic deficits (Hodges and Patterson, 2007). The semantic deficits could be associated with integration failure (e.g., Vallet et al., 2011b; Hoffman et al., 2014). The second and third hypotheses are explored by contrasting populations showing sensory and perceptual declines without and with multisensory integration deficits, respectively in normal aging and in AD. The dynamic integration hypothesis is tested by manipulating visual isolation (Hunt and Lamb, 2001). One-quarter of the items is associated with a distinctive yellow background that should increase memory performance in all groups (YA, HE, SD) except in AD.

In other words, young adults will represent the reference group of the present study. Compared to them, the concomitant decline in perception and cognition (including episodic memory) of the HE would illustrate how reduced perception might impact memory performance (reduced recall and confusion errors). HE will be the control group for AD and SD patients. Compared to HE, AD shall present significantly worst performances in all episodic memory tasks and shall also commit intrusion errors due to an integration deficit. AD shall be the only group to not benefit from the perceptual isolation. Finally, SD should exhibit relatively similar performance than HE on episodic memory tasks, with the exception of free recall tasks (naming deficit), whereas these patients should be the only group with major semantic deficits (matching and naming task).

## Method

### Participants

A total of 103 participants were included in the present study (see Table 1). These participants were divided into four groups: 40 young adults, 40 healthy elderly (HE), 20 patients with Alzheimer's disease (AD), and three patients with Semantic Dementia (SD). AD and SD patients received a diagnosis from a specialist (e.g., a registered neurologist). Diagnoses were confirmed during a consensus meeting between an AD's expert university professor, several neuropsychologists and a neurologist for the AD patients, with the addition of a speech language pathologist and an occupational therapist for the SD patients.

**Table 1:** Means (and standard deviations) for the demographic data for the young adults (YA), healthy elderly adults (HE), patients with Alzheimer's disease (AD) and patients with Semantic Dementia (SD).

	**Young adults (*n* = 40)**	**Healthy elderly (*n* = 40)**	**Alzheimer's disease (*n* = 20)**	**Semantic dementia (*n* = 3)**
Age	22.9 (3.3)	73.85 (5.8)	75.95 (6.4)	66 (12.5)
Gender (F/M)	28/12	28/12	14/6	21/2
Education (in years)	14.9 (2)	13.2 (4.4)	13.3 (4.2)	13.33 (1.2)

The AD patients received a diagnosis of probable Alzheimer's disease according to the Diagnostic and Statistical Manual of Mental Disorders–Fourth Edition (American Psychiatric Association, 2004) and the National Institute of Neurological and Communicative Disorders and Stroke–Alzheimer's Disease and Related Disorders Association (NINCDS-ADRDA) criteria (McKhann et al., 1984). They were in the early to moderate stages of the disease as defined by a MMSE score between 18 and 27. All SD patients received from a neurologist a diagnosis of probable Semantic Dementia (Neary et al., 1998). They exhibited a significant loss of word meaning and word-finding difficulties. They were in the early to moderate stages of the disease. Despite the number of SD patients seems very small, the disease is rare so that most of the previous publications on SD were done with unique or multiple cases.

AD patients were recruited in Quebec City (Quebec, Canada) in the community, in a community center, or in the pool of patients already followed in our laboratory. SD patients were recruited from a larger project conducted in Quebec City (see Auclair-Ouellet et al., 2016). Participants in the HE group were recruited through public announcements and in two community centers in Quebec City. Participants in YA group were recruited at Laval University and were matched for education and gender with participants in the HE and AD groups. In addition, participants in the HE group were also matched for age with the AD patients (see Table 1). Except the SD patients, about the two-third of the participants in each group (YA, HE, AD) took part in a larger study on memory (see Vallet et al., 2013).

Health information was gathered from all participants during an extensive medical history and neuropsychological interview (see Appendix in Supplementary Material for the detailed cognitive profile of each group). In addition, most of the participants also completed the NPI (NeuroPsychiatric Inventory, Cummings et al., 1994) (or completed by a relative in the case of patients with dementia). Participants with a medical history and/or taking medications for conditions with known sensory or neurological effects were excluded, such as schizophrenia, mild to severe head injury, epilepsy, alcohol or other drug abuse, and so on. Participants who reported a diagnosis of depression or anxiety were included only if they were stable on their medication and if they were non-symptomatic at the time of the study. All participants in the study were native French speakers and demonstrated adequate speech, visual and hearing performances.

All participants underwent a neuropsychological screening battery (see Table 1). Nonetheless, the tests completed varied according to the project in which the participant was originally involved. All participants completed a cognitive speed test [simple reaction time task (SRT)] and a standard test of general cognitive functioning [Mini-Mental State Examination (Folstein, 1975)]. Except for SD patients, they were all tested on verbal memory [(RL/RI-16 free and cued recall task Van der Linden, 2004)], executive functions [Trail Making Test (TMT, Delis et al., 2001; Lezak et al., 2004); and Stroop test (Godefroy et al., 2010)], and executive-semantic functions [word fluency test (Cardebat et al., 1990)].

### Material

The SEMEP test is based on the visual part of the PPTT in which participants must match semantically related pictures on 52 boards. On each board, three black-and-white line-drawn pictures are displayed as a triangle. On the top, one picture serves as a model (e.g., a pyramid). The two remaining pictures are at the base of the triangle. One picture is the target (e.g., a palm tree) and the other one represents a distractor (e.g., a pine tree).

**Figure 1:**
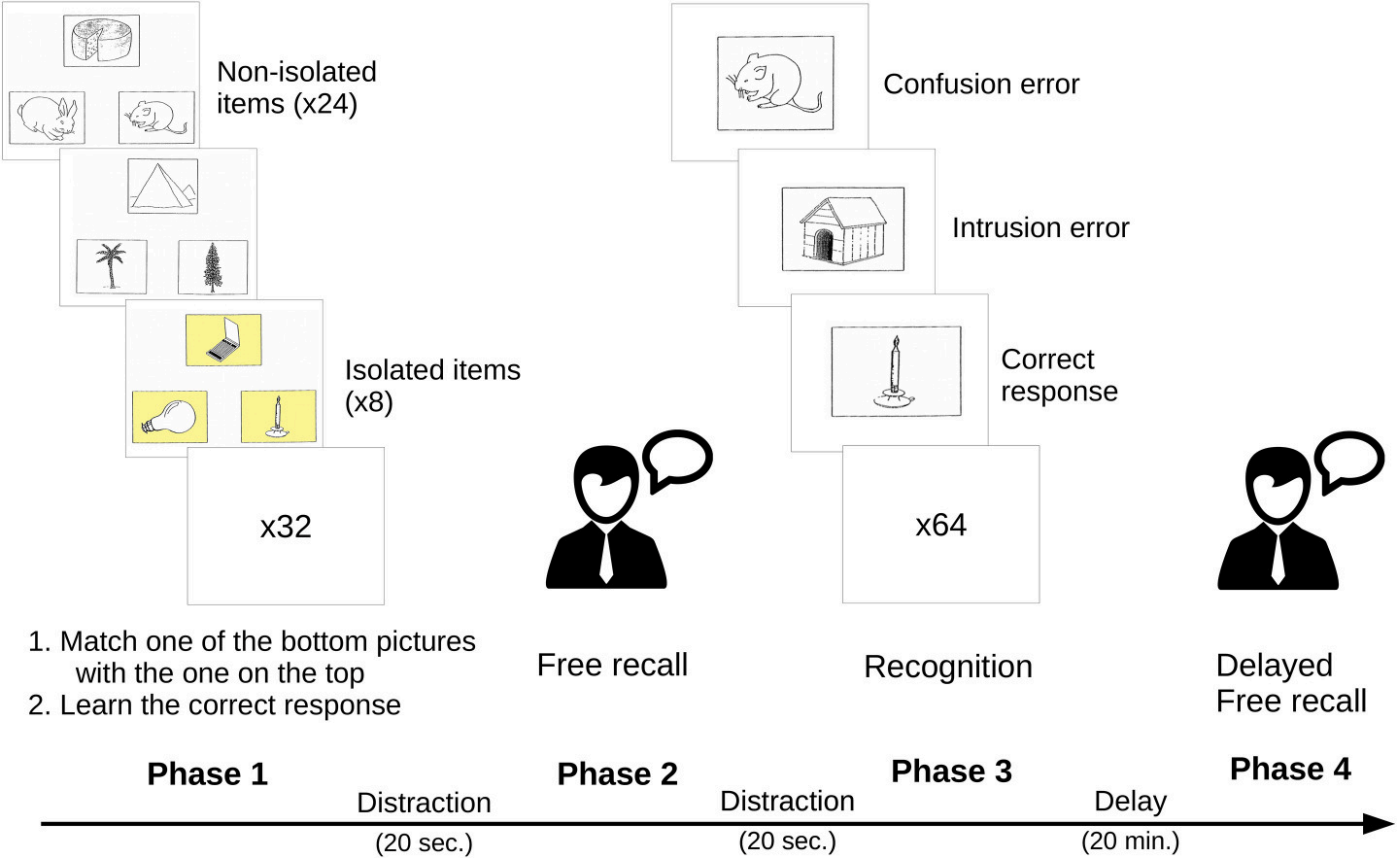
Illustration of the general procedure and of the material used in the SEMEP.

All the cards of the visual subset of the PPTT were scanned in order to create a numeric version that could be modified. In the encoding/matching phase, 32 of the 52 original cards were selected (cf. Table 2). Among these 32 cards, one-quarter (i.e., 8) were randomly chosen to be visually isolated. The isolation consisted in adding a yellow background to the three items on the board. The non-isolated items remained with the original white background. Illustration of the material used in presented in Figure 1.

**Table 2:** List of the stimuli included in the encoding/matching phase of the SEMEP.

	**Model**	**Item 1**	**Item 2**	**Isolation**
P1	Bottle	mug	**glass**	
P2	TV aerial	**television**	radio	
P3	Fork	ladle	**spoon**	
1	glasses	**eye**	ear	
2	Hands	**gloves**	slippers	Isolated
3	cheese	rabbit	**mouse**	
4	thimble	**needle**	thread	
5	saddle	goat	**horse**	
6	pillow	**bed**	chair	
7	trees	onion	**apple**	
8	matches	light bulb	**candle**	Isolated
9	pyramid	**palm tree**	pine tree	
10	bat	**owl**	woodpecker	
11	web	bee	**spider**	
12	tent	**fire**	radiator	
13	soldiers	church	**castle**	Isolated
14	caterpillar	**butterfly**	dragonfly	
15	nun	**convent**	house	Isolated
16	whool	dogs	**sheep**	
17	eggs	**hen**	swan	
18	puddle	sun	**clouds**	
19	fish	**cat**	dog	
20	drill	**screw**	nail	Isolated
21	stethoscope	tongue	**heart**	
22	logs	hammer	**saw**	Isolated
23	safety pin	girl	**baby**	
24	milk	**cow**	bull	
25	razor	**chin**	noze	Isolated
26	curtain	door	**window**	
27	rocket	star	**moon**	
28	mask	**clown**	mayor	
29	path	hands	**feet**	
30	ink	pencil	**pen**	
31	padlock	**bicycle**	car	Isolated
32	eskimo	rowing boat	**kayak**	

*Eight board were chosen to be isolated by the addition of a yellow background to the pictures in Figure 1. P1, P2, practice items; Bold items, correct matching responses and targets to be learned; Isolated, boards with a yellow background added*.

A first reason to decrease the number of cards was related to the nature of the material itself as some items were repeated on multiple boards. We thus selected the cards in order to avoid any double presentation of a given item. Furthermore, some associations were demonstrated as being inappropriate for the Quebec French-speaking population. For instance, some items were dramatically poorly processed compared to the others as the windmill–tulip (item 16) or the acorn-pig (item 40) associations (Callahan et al., 2010). The second reason was to decrease the cognitive load associated with the learning phase and to avoid a feeling of failure due to a large number of items to be learned. Finally, the last reason to reduce the number of stimuli regards the need to keep enough items from the original task to be used in the recognition task. The selection was also done to ensure that the correct matching responses were equally presented on each side of the card.

The foils used in the recognition task were selected to have similar visual characteristics than the targets. From the three-picture cards, each item is numerically isolated in order to create new cards with only one item on them. From these pictures, all the targets (items to learn in the encoding phase) were included (32), as well as the same number of foils (32). These foils were selected to be in two conditions: (1) old-foiled: 16 pictures that were presented in the encoding phase, but were not to be learned, (2) new-foiled: 16 pictures that were never presented in the encoding phase. Half of the old-foiled (8) was the distractor and the other half was the model on the original cards. These pictures have been printed and displayed on cards that are easy to manipulate. The order of presentation of the items was randomly defined, but was kept constant for all the participants (cf. Table 3).

**Table 3:** List of the stimuli included in the recognition task of the SEMEP.

	**Item**		**Item**		**Item**		**Item**
1	kennel	17	armchair	33	**fire**	49	**heart**
2	candle^**^	18	**window**	34	tulip	50	*igloo*
3	*car*	19	table	35	**owl**	51	**feet**
4	**spider**	20	*eskimo*	36	*light bulb*	52	screw^**^
5	*tent*	21	**bed**	37	**needle**	53	*radiator*
6	blackboard	22	*ink*	38	**eyes**	54	lamp
7	**baby**	23	worm	39	bus	55	bicycle^**^
8	**clouds**	24	*glasses*	40	castle^**^	56	matches
9	cage	25	**moon**	41	*hammer*	57	**apple**
10	**butterfly**	26	*nose*	42	gloves^**^	58	**pen**
11	battery	27	**palm tree**	43	**sheep**	59	*ear*
12	windmill	28	**kayak**	44	**horse**	60	chin^**^
13	couvent^**^	29	*razor*	45	carrot	61	**cat**
14	*pencil*	30	**mouse**	46	saw^**^	62	*cadlock*
15	anchor	31	ship	47	*logs*	63	desk
16	**clown**	32	ring	48	**cow**	64	**hen**

Bold items, targets; Italic items, confusion errors;

** *, isolated items*.

## Procedure and design

### General procedure

This research was approved by the Ethical Committee of the “Centre de recherche Université Laval Robert-Giffard” (project #228) and all participants signed an informed consent form before the experimental session started. Each participant was tested individually.

All participants completed the whole experimental protocol in one session of ~2 h. Following a short clinical interview, they were submitted to the cognitive battery. They first responded to the MMSE, and then to the RL/RI-16 memory test. During the 15 min separating the last recall from the delayed recall, they completed different non-verbal tests varying in function of the project (e.g., the simple reaction time, visual perception tasks). This was followed by the SEMEP and between the recognition and delayed free recall tasks (see below), they completed other non-verbal tests such as the TMT test.

### SEMEP-procedure

A general presentation of the SEMEP and of the different scores computed are provided in Table 4. The experimenter presented the test as a memory test based on pictures. It was highlighted that the participant had to learn a lot of items and it was clearly stated that these items had to be recalled later.

**Table 4:** Summary of the procedure used in the SEMEP with the different scores collected.

**Phase**	**Task**	**Description**	**Score**
Phase 1	Matching task	Match one of the bottom pictures to the top picture	Score /32
	Encoding task	Learn the right answer	
	*Distraction 1 (20 s.)*	*Countdown by step of one*	
Phase 2	Free recall task	Recall as much as possible words to be learned in Phase 1	Score /32
	*(2 min.)*	Intrusion errors (item never presented)	Number of errors
		Confusion errors (item saw, but not to learn)	Number of errors
		Isolation (items recalled that were isolated in Phase 1)	Score /8
	*Distraction 2 (20 s)*.	*Countdown by step of one*	
Phase 3	Naming task	Give the name of the object depicted on the card	Score /64
	Recognition task	Recognize the pictures corresponding to the correct matching in phase 1	Score /32
		Intrusion errors (item never presented)	Number of errors
		Confusion errors (item saw, but not to learn)	Number of errors
		Isolation (items recognized that were isolated in Phase 1)	Score /8
	*Delay (20 min.)*		
Phase 4	Delayed free recall task	Recall as much as possible words to be learned in Phase 1	Score /32
	*(2 min.)*	Intrusion errors (item never presented)	Number of errors
		Confusion errors (item saw, but not to learn)	Number of errors
		Isolation (item recalled that were isolated in Phase 1)	Score /8

During the encoding phase, the instructions insisted on the fact that two tasks had to be done at the same time: a matching task and a learning task. Participants were told about the matching task that “on each card, you will see three line-drawn pictures organized in a triangle. The picture on the top will be your model. Your first task will be to judge which one of the two pictures, presented at the bottom, best matches the model.” The first example was then introduced.

Once the participant had well understood how to proceed, the experimenter presented the learning task: “in addition to the matching task, I will ask you to learn the corresponding name of the correct answer of the matching task. Be careful, your task is to learn and remember only the correct answer. If you happen to recall the other pictures from the card, it will be considered as errors.” The two other practice cards were presented by stressing out that it was only a practice, so that the participant did not have to learn these examples.

If the participant had no question about the procedure, the first phase was then summarized: “for each card, you will first tell me which one of the bottom pictures matches the top picture. I will confirm or correct your answer if necessary. Then, I will always confirm which item you will have to learn.”

Each board was presented one at a time. The participant did not have any time constraint, but the experimenter tried to keep the presentation of the item relatively constant (~8 s per card). If the participant made a mistake, the experimenter corrected her/him immediately and provided the right answer. Synonyms were accepted and used by the experimenter in the learning instructions. For instance, if the participant said “fortress” instead of “castle,” the experimenter then stated: “that's correct, and you will have to learn “fortress.”

In order to avoid recency effect from short-term memory, participants had then to countdown from a random number (e.g., 326) by steps of one during 20 s. Once completed, the experimenter asked the participant to recall as much words as possible that were to be learned. It was highlighted to be careful to give only the correct answer and not the names of other pictures that would be considered as errors. The participant had 2 min to complete the task. If errors were committed during the recall task, they were not corrected.

A second distractor task was realized before the third phase. The same countdown task was chosen to start from a different number (e.g., 450). The third phase consisted of a yes/no visual recognition task combined with a naming task presenting one card showing one item at a time. For each card, participants were asked to name the depicted object, and then to indicate by a “Yes” or “No” response whether the picture was learned in the learning phase. Once again, it was stressed out that only the correct answers from the matching task had to be considered as a “Yes” response. When an error was made, the experimenter corrected the response.

No mention was made that a delayed free recall task would take place after a 20-min. delay. During this time, the other non-verbal tasks of the general protocol were completed as described in the previous section. At last, the delayed free recall task was completed using the same procedure than that used in the first free recall task.

In the first phase, the correct matching responses were recorded (on 32). In the recall tasks, the experimenter recorded the number of correct responses (items to be remembered, on 32) as well as the number of isolated items correctly recalled (on 8). In the recognition task, the number of correctly named items (on 64) as well as the number of correctly recognized items were noted (on 32) with the addition of the number of isolated items correctly recognized (on 8). In the free recall and recognition tasks, (1) a confusion error was defined as the recall or recognition of an item presented in the encoding phase that was not to be learned; (2) an intrusion error consisted in the recall or recognition of an item never presented to the participant in the encoding phase.

### Statistical analysis

The data was processed and analyzed using R version 3.3.1 (R Foundation for Statistical Computing). In addition to the raw scores recorded in the SEMEP, the proportion of isolated, confusion errors and intrusion errors were computed for the free recall and delayed free recall tasks. The proportion of isolated item recall was computed with the formula: $\frac{number\;of\;isolated\;items\;recalled}{total\;number\;of\;items\;recalled}$; and errors rate with the formula: $\frac{number\;of\;errors}{total\;number\;of\;items\;recalled}$. Analyses of variance (ANOVA) were conducted on each dependent variable with the Group (YA vs. HE vs. AD) as a between-subjects variable. SD patients were excluded from the analyses due to the too limited sample size (only three patients), but they were included in the z-scores profiling. Z-scores were computed for the mean scores of all the elderly groups using the data from the YA as a reference (z = $\frac{mean_{score}-mean_{YA}}{sd_{YA}}$, with mean_YA_ and sd_YA_ as the mean and standard deviation values of the young adults). In order to avoid infinite values, the mean and standard deviation values were replaced by the value of 0.1 when they equaled to 0. Subsequent comparisons were conducted using Tukey *post hoc* analyses. The common trace hypothesis was tested using a Pearson correlation analysis (bilateral) between the scores of the semantic tasks (matching and naming) and of the episodic tasks (free recall, recognition). An alpha level of 0.05 was used as a significant threshold for all the analyses.

## Results

As shown in Table 5, different patterns of results could be observed on the SEMEP as a function of the comparison underwent. First of all, HE, compared to YA, showed poorer performance on immediate and delayed free recall tasks, whereas recognition (correct scores) appeared preserved. Despite the fact that HE recalled fewer isolated items than YA, their proportion of recall of these items did not differ significantly from that of their younger counterparts. They also did not produce more intrusion errors than YA, but they did commit more confusion errors across the different tasks (recall, recognition and also on the proportions computed).

**Table 5:** Mean scores (and standard error) for all the raw and proportion of isolated items, confusion errors and intrusion errors for the young adults (YA), healthy elderly (HE), and patient with Alzheimer's disease (AD).

	**YA**	**HE**	**AD**	**SD**	**YA-HE**	**YA-AD**	**HE-AD**	***F***
**MATCHING**								
Correct matching	31.4 (0.24)	30.67 (0.24)	29.05 (0.34)	26.67 (4.16)	0.09	0	0	15.59^***^
**FREE RECALL**								
Correct not isolated	16.33 (0.73)	12.67 (0.73)	3.5 (1.03)	5.67 (2.08)	0	0	0	52.19^***^
Correct isolated	4.08 (0.24)	2.9 (0.24)	0.75 (0.34)	1.33 (1.53)	0	0	0	32.21^***^
Confusion errors	0.47 (0.14)	1.47 (0.14)	1.15 (0.2)	0 (0)	0	0.02	0.39	12.66^***^
Intrusions errors	0 (0.11)	0.05 (0.11)	1 (0.16)	0 (0)	0.95	0	0	14.83^***^
**DELAYED FREE RECALL**								
Correct not isolated	20.5 (0.8)	15.7 (0.8)	5.05 (1.14)	8 (1)	0	0	0	61.72^***^
Correct isolated	5.8 (0.24)	4.63 (0.24)	0.6 (0.34)	2.33 (2.08)	0	0	0	78.09^***^
Confusion errors	0.4 (0.17)	1.52 (0.17)	1.5 (0.24)	0.33 (0.58)	0	0	1	13.37^***^
Intrusions errors	0 (0.14)	0.05 (0.14)	2.1 (0.2)	0.33 (0.58)	0.97	0	0	41.95^***^
**RECOGNITION**								
Correct not isolated	30.75 (0.48)	29.65 (0.48)	23.85 (0.68)	29.67 (1.53)	0.24	0	0	36.68^***^
Correct isolated	7.77 (0.18)	7.17 (0.18)	4.85 (0.25)	7.33 (0.58)	0.05	0	0	46.96^***^
Confusion errors	0.22 (0.32)	1.65 (0.32)	6.2 (0.45)	2.33 (2.08)	0.01	0	0	60.27^***^
Intrusions errors	0 (0.38)	0.03 (0.38)	5.05 (0.53)	0 (0)	1	0	0	35.81^***^
**NAMING**								
Correct	63.25 (0.46)	62.67 (0.46)	57.05 (0.65)	52.67 (0.58)	0.65	0	0	33.5^***^
**PROPORTIONS**								
FR (isolated)	0.25 (0.02)	0.23 (0.02)	0.28 (0.03)	0.19 (0.19)	0.84	0.78	0.52	0.62
FR (confusions)	0.03 (0.02)	0.12 (0.02)	0.27 (0.04)	0 (0)	0.02	0	0	16.8^***^
FR (intrusions)	0 (0.01)	0 (0.01)	0.12 (0.02)	0 (0)	0.97	0	0	19.64^***^
DFR (isolated)	0.29 (0.02)	0.3 (0.02)	0.15 (0.02)	0.28 (0.25)	0.76	0	0	15.98^***^
DFR (confusions)	0.02 (0.01)	0.1 (0.01)	0.19 (0.02)	0.04 (0.07)	0	0	0	25.24^***^
DFR (intrusions)	0 (0.01)	0 (0.01)	0.22 (0.01)	0.03 (0.06)	0.97	0	0	97.97^***^

P, p-values computed for each ANOVA with the group (HE, aMCI, AD) as a between subject variable;

*** *p < 0.001; FR, free recall; DFR, delayed free recall*.

Secondly, AD patients did exhibit poorer performance than YA in all conditions except for the proportion of isolated items recalled in the immediate recall task. This might be explained by the limited number of items recalled by the AD patients (only 3.5 on average).

Finally, AD patients also showed poorer performances than HE on almost all scores except for the number of confusions. This result has to be moderated by the fact that compared with HE, AD patients committed more confusion errors, in proportion, in the two recall tasks.

The common trace hypothesis was also tested using a correlation analysis between the scores of the semantic tasks (matching and naming) and of the episodic tasks (free recall, recognition) as presented in Table 6. The analysis could not be conducted on the intrusion errors in the free recall tasks as there was not enough variance. Corroborating the hypothesis, the semantic scores were significantly associated with most of the episodic scores, excepted for the confusion errors in the recall tasks.

**Table 6:** Correlation analysis between the semantic (matching and naming) scores and the episodic (free recall, recognition, errors) scores of the SEMEP across all groups of participants (young adults, healthy elderly and patients with Alzheimer's disease).

**Semantic**		**Matching**	**Naming**
**EPISODIC**			
FR	Corr.	0.29^**^	0.51^***^
	Corr. Isolated	0.33^***^	0.45^***^
	Confusion Err.	−0.15	0.07
Delayed FR	Corr.	0.26^**^	0.48^***^
	Corr. Isolated	0.34^***^	0.56^***^
	Confusion Err.	−0.17	−0.18
Recognition	Corr.	0.22^*^	0.32^***^
	Corr. Isolated	0.43^***^	0.35^***^
	Confusion Err.	−0.37^***^	−0.63^***^
	Intrusion Err.	−0.54^***^	−0.59^***^

FR, free recall; Corr., correct; Err., errors; VOSP, visual object and space perception;

* p < 0.05;

** p < 0.01;

*** *p < 0.001; Bold items, significant correlation*.

## Discussion

The aim of the present article was to assess a new memory test, the SEMEP, that respect some core assumptions of embodiment in the field of memory, i.e., (1) the common memory traces between semantic and episodic knowledge, (2) the grounding of knowledge into its sensory-motor components, (3) the dynamic integration of knowledge to emerge as episodic memories. Thus, hypotheses associated with these assumptions were tested across four different populations.

It was first hypothesized that HE compared to YA would present reduced recall performances on the SEMEP. As expected, the results showed that HE recalled statistically fewer items than YA in the immediate and delayed recall tasks. However, their performance was not clinically impaired (z-scores deviation inferior to 1), which might be explained by the visual nature of the material used in the SEMEP. Different studies have indeed shown that HE benefit from visual material to be learned compared to verbal one (e.g., Smith et al., 2015).

It was also hypothesized that HE compared to YA would make only more confusion errors. The results supported this hypothesis. HE committed more confusion errors than YA, but did not make more intrusion errors than YA in all conditions of the SEMEP. These findings are supported by the results of previous reports that have demonstrated that HE are more vulnerable to memory errors, especially to false alarms, than their younger counterparts (see Devitt and Schacter, 2016 for a recent review). However, HE do not falsely recognize stimuli that are not closely related to the target (e.g., Toner et al., 2009). There are different hypotheses in the literature to account for the confusion errors made by HE. The most commonly admitted hypotheses rely on the deficits of executive functions in aging, in line with the alteration of their frontal lobes (e.g., Butler et al., 2004; but see Chan and McDermott, 2007 for a different point of view). For instance, according to the source-monitoring hypothesis, false memories occur when a person is not able to track the source of the stimulus as being old or new (Johnson et al., 1993). Participants need to retrieve specific characteristics from the event that will help them to make a decision about its source. Because these effortful and strategic processes are impaired in aging, HE commit more false alarms than YA (e.g., Meade et al., 2012).

From an embodied perspective, the increase memory errors in HE might be explained by the degradation of perception in aging. It has been known for a long time that the sensory/perceptual decline in healthy aging is associated with the cognitive decline (see Roberts and Allen, 2016 for a recent review). Growing evidence especially shows that memory and perception are very tightly bound (e.g., Graham et al., 2010; Rey et al., 2015; see Appendix B in Supplementary Material for data on a subset of the HE group), and that memory traces in HE are grounded in their sensory-motor components, as in YA (e.g., Vallet et al., 2011a, 2013). Therefore, degraded perceptual processing, as in HE, should directly impoverish their memory traces (Humes et al., 2013; Vallet, 2015). The degraded memory traces should, in turn, decrease the distinctiveness of the memory traces, and consequently, it shall be more difficult to distinguish one memory trace from another (see Brunel et al., 2013; Vallet et al., 2016b). In other words, HE should commit more confusion errors as they require more pronounced distinctive features to correctly reject a related lure (Butler et al., 2010).

The distinctiveness heuristic (see Dodson and Schacter, 2002) appears also a useful hypothesis to interpret the results observed for the isolated items in the present study. HE benefited from the visual isolation in a similar fashion than YA (i.e., both groups obtained similar proportions of correct free recall on isolated items; see the bottom of Table 5) (see also Geraci et al., 2009). Isolation is a method commonly used to enhance the distinctiveness of a small set of items to be learned by giving them a particularity not shared with the other items (e.g., Brunel et al., 2010). Despite the altered perception observed in aging, HE shall have preserved, and perhaps enhanced, multisensory integration (Laurienti et al., 2006). This might explain the present result.

Otherwise, the results of the HE group showed performances relatively similar to those of YA on the semantic and recognition tasks. Numerous studies have found preserved, and sometimes enhanced, semantic memory in aging (e.g., Nyberg et al., 2003; see Park and Gutchess, 2002 for a review). It is also frequent to observe similar, or slightly impaired, recognition performance for HE when the task is not too demanding (Danckert and Craik, 2013; Koen and Yonelinas, 2014).

**Figure 2:**
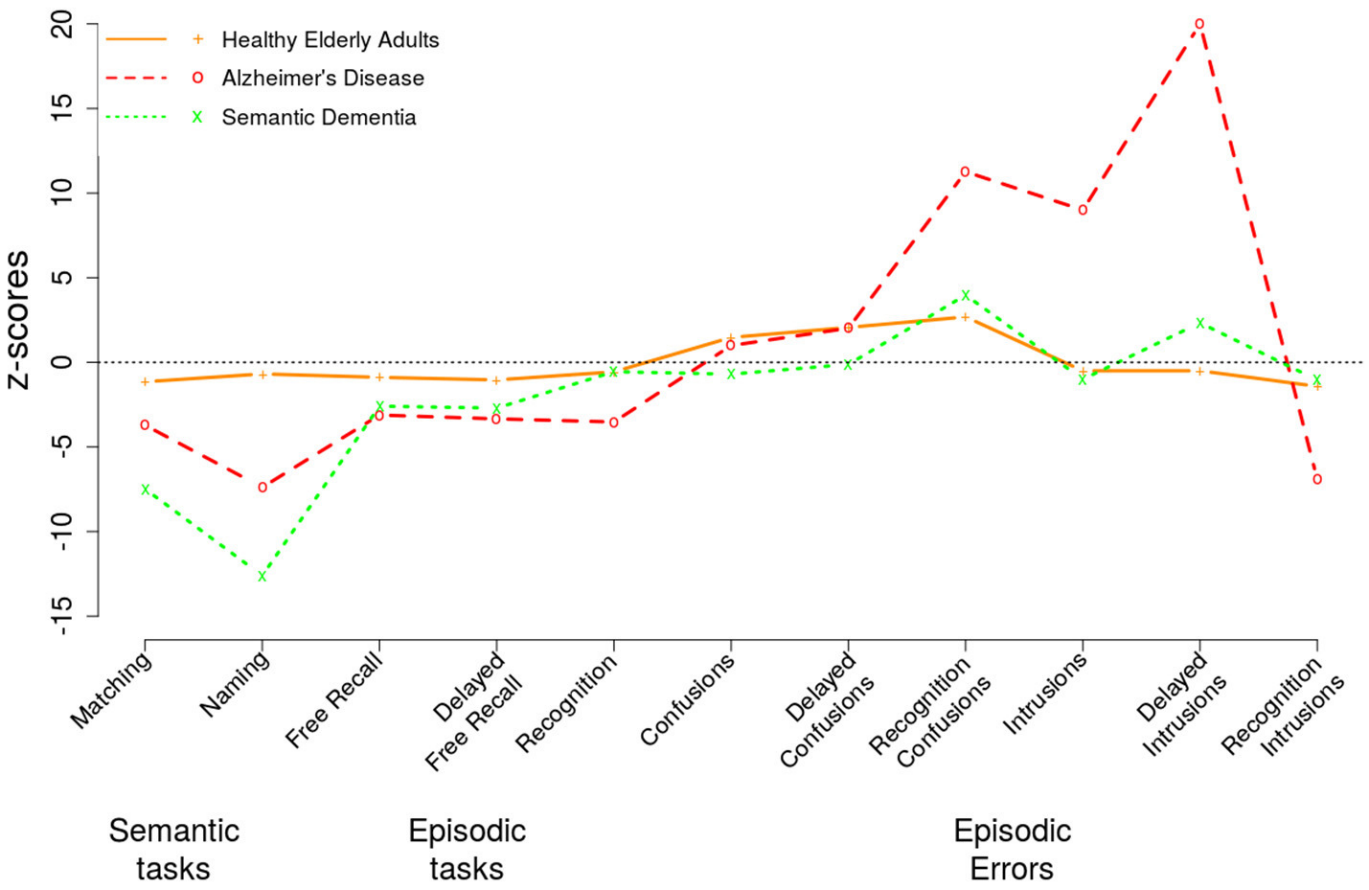
z-scores of healthy elderly adults, patients with Alzheimer's disease and patients with semantic dementia using data from the young adults as reference across the main scores of the SEMEP.

As expected, AD patients showed severe deficits in episodic memory across the tasks. AD is characterized with severe episodic memory disorders together with a disconnection syndrome (see Delbeuck et al., 2003). It could thus be expected that these patients will show more marked memory impairments, which will expand to memory errors for unrelated content, as well as a deficit to integrate supplementary perceptual information (in the isolation procedure). Nonetheless, AD correctly recalled a relatively similar number of items than SD patients (see Figure 2). This pattern has been previously reported in the literature, especially for the recall of pictures, on the contrary to verbal memory that is more impaired in AD (Scahill et al., 2005). AD patients also produced a similar amount of confusion errors than HE in the recall tasks. However, the proportions of confusion errors in the recall tasks and the false alarms in recognition were significantly more important for AD patients compared to HE.

Interestingly, AD patients were the only group to commit intrusion errors compared to the other groups. These errors were produced both in the recall and recognition tasks. Despite intrusion errors are typically underlied by frontal impairment, such as the one seen in dementia with Lewy bodies (Doubleday and Snowden, 2002), some studies have found a similar pattern of intrusion errors between AD and fronto-temporal dementia (Pasquier and Grymonprez, 2001). These errors could indicate the weakness of AD's memory traces compared to those of HE (see Vallet et al., 2016b). The overall pattern of performance observed in the present study is yet consistent with other published studies (e.g., Greenaway et al., 2006).

Finally, the isolation procedure let emerges an interesting result. Whereas, all groups of participants appeared to benefit from the increase distinctiveness provided by this procedure, AD patients did not. All scores for these items, except for the proportions of isolated items recalled in the immediate recall task, were significantly lower in AD compared to those observed in YA and HE. Moreover, the probability that AD would recall (in the delayed task) and recognize the isolated items was in fact inferior to chance, which was here at 25%^1^. The fact that this deleterious effect of isolation was not observed in the immediate free recall might be due to either the very limited number of items recalled then (3.5 in average compared to 5.05 in the delayed recall), or to the accelerate forgetting reported in AD (e.g., Estévez-González et al., 2003).

This result might be surprising in the traditional multisystem memory approach, but it is expected in embodied cognition theories. Indeed, this deleterious effect is unlikely coming from (1) a specific visuoperceptual decline in AD patients, as they exhibit preserved visual repetition priming (e.g., Fleischman, 2007); (2) from a specific deficit in the isolation effect as isolation effect appears less efficient in AD, but still beneficial, when it is the font size that is manipulated (Vitali et al., 2006) (3) from an overload of their cognitive resources since the simple addition of visual information, such as a background, is not sufficient to impair cognitive performance in AD (see Vallet et al., 2013). In the present study, the yellow background appears as a burden which is likely constitutes supplementary information to be bound within the memory trace (see Versace et al., 2014). Indeed, according to the embodied cognition theories, AD show impaired multisensory integration (Delbeuck et al., 2007), which could be related to their disconnection syndrome (Vallet et al., 2013). The disconnection syndrome in AD might explain why these patients present memory deficits in a situation which requires the dynamic interplay between sensory (or multidmodal) components (Festa et al., 2005). This hypothesis remains to be further explored to determine how the integration deficit may play a central role in episodic memory impairment (see also Buschke et al., 2017).

Finally, SD patients showed severe deficits in the semantic memory tasks (matching and naming) as expected in this population Hodges and Patterson, 2007). They also presented reduced free recall performance which appeared to be relatively similar to AD. Nonetheless, this latter finding should be discussed in the light of their naming deficits (Graham et al., 2000). Indeed, SD patients performed well on recognition, comparable to HE, which suggests a relative preservation of episodic memory compared to AD. They also committed a similar amount of confusion and intrusion errors than HE. This suggests that their episodic memory was not as affected by other integration deficits as in AD.

Thus, the difficulties faced by SD on free recall tasks may be due to the difficulty of finding their words rather than remembering the words. This hypothesis is supported by the clinical experience during the study; SD patients did try very hard to find the words of items to be recalled, and then abandoned to recall (i.e., name) another item. Of course, these findings should be interpreted with caution as only three patients were included in the present study. A greater number of patients should be included in future studies before drawing any conclusion. Yet, SD patients were included in the present study in order to illustrate the difference of performance pattern between the elderly groups rather than to provide strong evidence in favor of their semantic and episodic patterns of performance.

Taken all together, the data of this study showed very distinctive patterns of performance between the elderly groups, as illustrated with the z-scores in Figure 2. It seems that confusion errors are common across all the elderly groups, with and without cognitive disorders. These errors are supposed to reflect the degradation of the memory traces so that they become less distinctive with aging. According to embodiment theories, this degradation results from the perceptual decline reported in aging. AD was the only group in the present study that exhibited regular intrusion errors. The intrusion errors could be interpreted as an integration deficit, which is also supported by the detrimental effect of the visual isolation in this group. This difference in the pattern of errors between the elderly and dementia groups emphasizes the need to further consider the type of errors to differentiate clinical population (e.g., Rouleau et al., 2001). Finally, SD patients exhibited the most severe semantic impairment compared to the other groups. They also recalled a few items, as AD patients, but for a different reason. As all episodic scores were reduced in AD patients, SD patients performed similarly to HE on the recognition task and committed the same type and number of errors as HE.

To conclude, the SEMEP seems to be an interesting tool to evaluate memory functioning in aging. Beyond the question of embodiment, the test permitted to show specific patterns of results for each group included in the study. In the future, studies may apply the principles used in this test to assess patients with different clinical diagnoses and in different situations to confirm the usefulness of the SEMEP in clinical settings.

## Author contributions

GV designed and conducted the study as well as analyzed the data. RV and MS helped to design the protocol and to analyze the data. CH, JM, and NB helped to conduct the study. All authors were significantly involved in the redaction of the manuscript.

### Conflict of interest statement

The authors declare that the research was conducted in the absence of any commercial or financial relationships that could be construed as a potential conflict of interest.

## Footnotes

^1^In recognition: *t*(19) = 3.51, *p* < 0.01. In delayed free recall: *t*(18) = 2.34, *p* < 0.05. Two-sided *t*-tests.

## References

[B1] Abe N. Fujii T. Nishio Y. Iizuka O. Kanno S. Kikuchi H.. (2011). False item recognition in patients with Alzheimer's disease. Neuropsychologia 49, 1897–1902. 10.1016/j.neuropsychologia.2011.03.015 21419789

[B2] American Psychiatric Association (2004). DSM-IV-TR Manuel Diagnostique et Statistique des Troubles Mentaux. Paris: Masson. 3787052

[B3] Auclair-Ouellet N. Macoir J. Laforce R. Bier N. Fossard M. (2016). Regularity and beyond: impaired production and comprehension of inflectional morphology in semantic dementia. Brain Lang. 155–156, 1–11. 10.1016/j.bandl.2016.02.002 26994740

[B4] Borghi A. (2015). An embodied and grounded perspective on concepts, in Epistemology of Ordinary Knowledge, eds Bianca M. Piccari P. (Cambridge: Scholar), 181–194.

[B5] Brunel L. Goldstone R. L. Vallet G. T. Riou B. Versace R. (2013). When seeing a dog activates the bark: multisensory generalization and distinctiveness effects. Exp. Psychol. 60, 100–112. 10.1027/1618-3169/a000176 23047916

[B6] Brunel L. Oker A. Riou B. Versace R. (2010). Memory and consciousness: trace distinctiveness in memory retrievals. Conscious. Cogn. 19, 926–937. 10.1016/j.concog.2010.08.006 20932777

[B7] Brunel L. Vallet G. Riou B. Rey A. Versace R. (2015). Grounded conceptual knowledge emergence from sensorimotor interactions, in Conceptual and Interactive Embodiment: Foundations of Embodied Cognition, eds Fisher M. Coello Y. (New York, NY: Psychology Press), 108–124.

[B8] Buschke H. Mowrey W. B. Ramratan W. S. Zimmerman M. E. Loewenstein D. A. Katz M. J.. (2017). Memory binding test distinguishes amnestic mild cognitive impairment and dementia from cognitively normal elderly. Arch. Clin. Neuropsychol. 32, 29–39. 10.1093/arclin/acx046 27680087 PMC5860053

[B9] Butler K. M. McDaniel M. A. Dornburg C. C. Price A. L. Roediger H. III. (2004). Age differences in veridical and false recall are not inevitable: the role of frontal lobe function. Psychon. Bull. Rev. 11, 921–925. 10.3758/BF03196722 15732704

[B10] Butler K. M. McDaniel M. A. McCabe D. P. Dornburg C. C. (2010). The influence of distinctive processing manipulations on older adults' false memory. Neuropsychology 17, 129–159. 10.1080/13825580903029715 19642045

[B11] Callahan B. Macoir J. Hudon C. Bier N. Chouinard N. Cossette-Harvey M.. (2010). Normative data for the pyramids and palm trees test in the Quebec-French population. Arch. Clin. Neuropsychol. 25, 212–217. 10.1093/arclin/acq013 20197296

[B12] Campbell K. L. Trelle A. Hasher L. (2014). Hyper-binding across time: age differences in the effect of temporal proximity on paired-associate learning. J. Exp. Psychol. Learn. Mem. Cogn. 40, 293–299. 10.1037/a0034109 23937237

[B13] Cardebat D. Doyon B. Puel M. Goulet P. (1990). Evocation lexicale formelle et sémantique chez des sujets normaux. Performance et dynamiques de production en fonction du sexe, de l'âge et du niveau culturel. Acta Neurol. Belg. 90, 207–217. 2124031

[B14] Casasanto D. (2011). Different bodies, different minds: the body specificity of language and thought. Curr. Dir. Psychol. Sci. 20, 378–383. 10.1177/0963721411422058

[B15] Chan J. C. K. McDermott K. B. (2007). The effects of frontal lobe functioning and age on veridical and false recall. Psychon. Bull. Rev. 14, 606–611. 10.3758/BF03196809 17972721

[B16] Cummings J. L. Mega M. Gray K. Rosenberg-Thompson S. Carusi D. Gornbein J. (1994). The neuropsychiatric inventory: comprehensive assessment of psychopathology in dementia. Neurology 44, 2308–2314. 10.1212/WNL.44.12.2308 7991117

[B17] Danckert S. L. Craik F. I. (2013). Does aging affect recall more than recognition memory? Psychol. Aging 28, 902–909. 10.1037/a0033263 23978011

[B18] Delbeuck X. Collette F. Van der Linden M. (2007). Is Alzheimer's disease a disconnection syndrome? Evidence from a crossmodal audio-visual illusory experiment. Dementia 45, 3315–3323. 10.1016/j.neuropsychologia.2007.05.001 17765932

[B19] Delbeuck X. Van der Linden M. Collette F. (2003). Alzheimer's disease as a disconnection syndrome? Neuropsychol. Rev. 13, 79–92. 10.1023/A:1023832305702 12887040

[B20] Delis D. Kaplan E. Kramer J. (2001). D-KEFS Executive Function System. San Antonio: The Psychological Corporation, a Harcourt Assessment Company.

[B21] Devitt A. L. Schacter D. L. (2016). False memories with age: neural and cognitive underpinnings. Neuropsychologia 91, 346–359. 10.1016/j.neuropsychologia.2016.08.030 27592332 PMC5075259

[B22] Dodson C. S. Schacter D. L. (2002). Aging and strategic retrieval processes: reducing false memories with a distinctiveness heuristic. Psychol. Aging 17, 405–415. 10.1037/0882-7974.17.3.405 12243382

[B23] Doubleday E. Snowden J. J. S. (2002). Qualitative performance characteristics differentiate dementia with Lewy bodies and Alzheimer's disease. J. Neurol. Neurosurg. Psychiatr. 72, 602–607. 10.1136/jnnp.72.5.602 11971046 PMC1737879

[B24] Estévez-González A. Kulisevsky J. Boltes A. Otermín P. García-Sánchez C. (2003). Rey verbal learning test is a useful tool for differential diagnosis in the preclinical phase of Alzheimer's disease: comparison with mild cognitive impairment and normal. Int. J. Geriatr. Psychiatry 18, 1021–1028. 10.1002/gps.1010 14618554

[B25] Festa E. Insler R. Salmon D. P. Paxton J. Hamilton J. Heindel W. C. (2005). Neocortical disconnectivity disrupts sensory integration in Alzheimer's disease. Neuropsychology 19, 728–738. 10.1037/0894-4105.19.6.728 16351348

[B26] Fleischman D. A. (2007). Repetition priming in aging and Alzheimer's disease: an integrative review and future directions. Cortex 43, 889–897. 10.1016/S0010-9452(08)70688-9 17941347

[B27] Fleischman D. A. Gabrieli J. D. E. (1999). Long-term memory in Alzheimer's disease. Curr. Opin. Neurobiol. 9, 240–244. 10.1016/S0959-4388(99)80034-8 10322182

[B28] Folstein M. (1975). “Mini-mental state.” A practical method for grading the cognitive state of patients for the clinician. J. Psychiatr. Res. 12, 189–198. 10.1016/0022-3956(75)90026-6 1202204

[B29] Geraci L. McDaniel M. A. Manzano I. Roediger H. L. (2009). The influence of age on memory for distinctive events. Mem. Cognit. 37, 175–180. 10.3758/MC.37.2.175 19223567

[B30] Glenberg A. M. Witt J. K. Metcalfe J. (2013). From the revolution to embodiment: 25 years of cognitive psychology. Perspect. Psychol. Sci. 8, 573–585. 10.1177/1745691613498098 26173215

[B31] Godefroy O. Azouvi P. Robert P. Roussel M. LeGall D. Meulemans T. (2010). Dysexecutive syndrome: diagnostic criteria and validation study. Ann. Neurol. 68, 855–864. 10.1002/ana.22117 21194155

[B32] Graham K. S. Barense M. D. Lee A. C. H. (2010). Going beyond LTM in the MTL: a synthesis of neuropsychological and neuroimaging findings on the role of the medial temporal lobe in memory and perception. Neuropsychologia 48, 831–853. 10.1016/j.neuropsychologia.2010.01.001 20074580

[B33] Graham K. S. Simons J. S. Pratt K. H. Patterson K. Hodges J. R. (2000). Insights from semantic dementia on the relationship between episodic and semantic memory. Neuropsychologia 38, 313–324. 10.1016/S0028-3932(99)00073-1 10678697

[B34] Greenaway M. C. Lacritz L. H. Binegar D. Weiner M. F. Lipton A. Munro Cullum C. (2006). Patterns of verbal memory performance in mild cognitive impairment, Alzheimer disease, and normal aging. Cogn. Behav. Neurol. 19, 79–84. 10.1097/01.wnn.0000208290.57370.a3 16783130

[B35] Greenberg D. L. Verfaellie M. (2010). Interdependence of episodic and semantic memory: evidence from neuropsychology. J. Int. Neuropsychol. Soc. 16, 748–753. 10.1017/S1355617710000676 20561378 PMC2952732

[B36] Hintzman D. L. (1990). Human learning and memory: connections and dissociations. Annu. Rev. Psychol. 41, 109–319. 10.1146/annurev.ps.41.020190.000545 2407168

[B37] Hodges J. R. Patterson K. (2007). Semantic dementia: a unique clinicopathological syndrome. Lancet Neurol. 6, 1004–1014. 10.1016/S1474-4422(07)70266-1 17945154

[B38] Hoffman P. Evans G. A. L. Lambon Ralph M. A. (2014). The anterior temporal lobes are critically involved in acquiring new conceptual knowledge: evidence for impaired feature integration in semantic dementia. Cortex 50, 19–31. 10.1016/j.cortex.2013.10.006 24268323 PMC3884130

[B39] Howard D. Patterson K. (1992). Pyramids and Palm Trees: a Test of Semantic Access from Pictures and Words. Bury St. Edmunds: Thames Valley Test Company.

[B40] Humes L. E. Busey T. A. Craig J. Kewley-Port D. (2013). Are age-related changes in cognitive function driven by age-related changes in sensory processing? Atten. Percept. Psychophys. 75, 508–524. 10.3758/s13414-012-0406-9 23254452 PMC3617348

[B41] Hunt R. R. Lamb C. A. (2001). What causes the isolation effect? J. Exp. Psychol. Learn. Mem. Cogn. 27, 1359–1366. 10.1037/0278-7393.27.6.1359 11713872

[B42] Johnson M. K. Hashtroudi S. Lindsay D. S. (1993). Source monitoring. Psychol. Bull. 114, 3–28. 10.1037/0033-2909.114.1.3 8346328

[B43] Koen J. D. Yonelinas A. P. (2014). The effects of healthy aging, amnestic mild cognitive impairment, and Alzheimer's disease on recollection and familiarity: a meta-analytic review. Neuropsychol. Rev. 24, 332–354. 10.1007/s11065-014-9266-5 25119304 PMC4260819

[B44] Laurienti P. J. Burdette J. H. Maldjian J. A. Wallace M. T. (2006). Enhanced multisensory integration in older adults. Neurobiol. Aging 27, 1155–1163. 10.1016/j.neurobiolaging.2005.05.024 16039016

[B45] Lezak M. Howieson D. Loring D. Fisher J. (2004). Neuropsychological Assessment 4th Edn. New York, NY: Oxford University Press.

[B46] MacDuffie K. E. Atkins A. S. Flegal K. E. Clark C. M. Reuter-Lorenz P. A. (2012). Memory distortion in Alzheimer's disease: deficient monitoring of short- and long-term memory. Neuropsychology 26, 509–516. 10.1037/a0028684 22746309 PMC3389800

[B47] McKhann G. Drachman D. Folstein M. Katzman R. Price D. Stadlan E. M. (1984). Clinical diagnosis of Alzheimer's disease: report of the NINCDS-ADRDA Work Group^*^ under the auspices of department of health and human services task force on Alzheimer's disease. Neurology 34:939. 10.1212/WNL.34.7.939 6610841

[B48] Meade M. L. Geraci L. D. Roediger I. I. I. H.. (2012). Neuropsychological status in older adults influences susceptibility to false memories. Am. J. Psychol. 125, 449–467. 10.5406/amerjpsyc.125.4.0449 23350303

[B49] Neary D. Snowden J. S. Gustafson L. Passant U. Stuss D. Black S. E. (1998). Frontotemporal lobar degeneration: a consensus on clinical diagnostic criteria. Neurology 53:1158 10.1212/WNL.51.6.1546 9855500

[B50] Nyberg L. Maitland S. B. Rönnlund M. Bäckman L. Dixon R. A. Wahlin A.. (2003). Selective adult age differences in an age-invariant multifactor model of declarative memory. Psychol. Aging 18, 149–160. 10.1037/0882-7974.18.1.149 12641319

[B51] Okado Y. Stark C. (2003). Neural processing associated with true and false memory retrieval. Cogn. Affect. Behav. Neurosci. 3, 323–334. 10.3758/CABN.3.4.323 15040552

[B52] Oker A. Versace R. Ortiz L. (2009). Spatial distinctiveness effect in categorisation. Eur. J. Cogn. Psychol. 21, 971–979. 10.1080/09541440802547567

[B53] Park D. C. Gutchess A. H. (2002). Aging, cognition, and culture: a neuroscientific perspective. Neurosci. Biobehav. Rev. 26, 859–867. 10.1016/S0149-7634(02)00072-6 12470698

[B54] Pasquier F. Grymonprez L. (2001). Memory impairment differs in frontotemporal dementia and Alzhemier's disease. Neurocase 7, 161–171. 10.1093/neucas/7.2.161 11320163

[B55] Rémy F. Vayssière N. Saint-Aubert L. Barbeau E. Pariente J. (2015). White matter disruption at the prodromal stage of Alzheimer's disease: relationships with hippocampal atrophy and episodic memory performance. Neuroimage 7, 482–492. 10.1016/j.nicl.2015.01.014 25685715 PMC4326466

[B56] Rey A. E. Vallet G. T. Riou B. Lesourd M. Versace R. (2015). Memory plays tricks on me: perceptual bias induced by memory reactivated size in Ebbinghaus illusion. Acta Psychol. 161, 104–109. 10.1016/j.actpsy.2015.08.011 26372936

[B57] Roberts K. L. Allen H. A. (2016). Perception and cognition in the ageing brain: a brief review of the short- and long-term links between perceptual and cognitive decline. Front. Aging Neurosci. 8:39. 10.3389/fnagi.2016.00039 26973514 PMC4772631

[B58] Rouleau I. Imbault H. Laframboise M. Bedard M. (2001). Pattern of intrusions in verbal recall: Comparison of Alzheimer's disease, Parkinson's disease, and frontal lobe dementia. Brain Cogn. Tennet. 11, 244–249. 10.1016/S0278-2626(01)80076-2 11527341

[B59] Scahill V. L. Hodges J. R. Graham K. S. (2005). Can episodic memory tasks differentiate semantic dementia from Alzheimer's disease? Neurocase 11, 441–451. 10.1080/13554790500287734 16393758

[B60] Schmidt S. R. (1991). Can we have a distinctive theory of memory? Mem. Cognit. 19, 523–542. 10.3758/BF03197149 1758300

[B61] Smith R. E. Hunt R. R. Dunlap K. R. (2015). Why do pictures, but not visual words, reduce older adults' false memories? Psychol. Aging 30, 647–655. 10.1037/pag0000044 26213799 PMC4556567

[B62] Toner C. K. Pirogovsky E. Kirwan C. B. Gilbert P. E. (2009). Visual object pattern separation deficits in nondemented older adults. Learn. Mem. 16, 338–342. 10.1101/lm.1315109 19403797

[B63] Tulving E. (1995). Organization of memory: Quo Vadis? in The Cognitive Neurosciences, ed Gazzaniga M. (Cambridge, MA: MIT Press), 839–847.

[B64] Vallet G. T. (2015). Embodied cognition of aging. Front. Psychol. 6:463. 10.3389/fpsyg.2015.00463 25932019 PMC4399201

[B65] Vallet G. T. Brunel L. Versace R. (2010). The perceptual nature of the cross-modal priming effect: arguments in favor of a sensory-based conception of memory. Exp. Psychol. 57, 376–382. 10.1027/1618-3169/a000045 20178946

[B66] Vallet G. T. Brunel L. Riou B. Vermeulen N. (2016a). Editorial: dynamics of sensorimotor interactions in embodied cognition. Front. Psychol. 6:1929. 10.3389/fpsyg.2015.01929 26779066 PMC4700472

[B67] Vallet G. T. Rouleau I. Benoit S. Langlois R. Barbeau E. J. Joubert S. (2016b). Alzheimer's disease and memory strength: gradual decline of memory traces as a function of their strength. J. Clin. Exp. Neuropsychol. 3395, 1–13. 10.1080/13803395.2016.1147530 27023396

[B68] Vallet G. T. Simard M. Versace R. (2011a). Sensory-dependent knowledge in young and elderly adults: arguments from the cross-modal priming effect. Curr. Aging Sci. 4, 137–149. 10.2174/1874609811104020137 21235495

[B69] Vallet G. T. Simard M. Fortin C. Versace R. Mazza S. (2011b). L'altération des connaissances sémantiques est-elle liée à une altération du traitement perceptif? Étude des atteintes catégories-spécifiques dans la démence sémantique. Geriatr. Psychol. Neuropsychiatr. Vieil. 9, 327–335. 10.1684/pnv.2011.0276 21896435

[B70] Vallet G. T. Simard M. Versace R. Mazza S. (2013). The perceptual nature of audiovisual interactions for semantic knowledge in young and elderly adults. Acta Psychol. 143, 253–260. 10.1016/j.actpsy.2013.04.009 23684850

[B71] Van der Linden M. (2004). L'épreuve de rappel libre/rappel indicé à 16 items (RL/RI-16), in L'évaluation des Troubles de La Mémoire, eds Van der Linden M. Coyette F. (Marseille: Solal), 25–47.

[B72] Versace R. Vallet G. T. Brunel L. Riou B. Lesourd M. Labeye E. (2014). ACT-IN: an integrated view of memory mechanisms. J. Cogn. Psychol. 26, 280–306. 10.1080/20445911.2014.892113

[B73] Vitali P. Minati L. Chiarenza G. Brugnolo A. Girtler N. Nobili F.. (2006). The Von Restorff effect in ageing and Alzheimer's disease. Neurol. Sci. 27, 166–172. 10.1007/s10072-006-0662-3 16897628

[B74] Waldie B. D. Kwong S. T. (2003). Remembering words never presented: false memory effects in dementia of the Alzheimer type. Neuropsychol. Dev. Cogn. B Aging Neuropsychol. Cogn. 10, 281–297. 10.1076/anec.10.4.281.28969

[B75] Yeung L. Ryan J. Cowell R. A. Barense M. D. (2013). Recognition memory impairments caused by false recognition of novel objects. J. Exp. Psychol. Gen. 142, 1384–1397. 10.1037/a0034021 23937183

[B76] Zimmer H. Mecklinger A. Lindenberger U. (2006). Handbook of Binding and Memory: Perspectives from Cognitive Neuroscience. Oxford: Oxford University Press.

