# Supplementary material for: A SEMantic and EPisodic Memory Test (SEMEP) Developed within the Embodied Cognition Framework: Application to Normal Aging, Alzheimer's Disease and Semantic Dementia
Source: Front Psychol. 2017 Sep 13;8:1493. doi: 10.3389/fpsyg.2017.01493 (PMC5601419; doi:10.3389/fpsyg.2017.01493)
Supplement: Supplementary file 1 [file Table1.docx]

| Table A: Demographic and neuropsychological data for the young adults (YA), healthy elderly adults (HE), patients with Alzheimer’s disease (AD) and patients with Semantic Dementia (SD). | | | | | | | |
| --- | --- | --- | --- | --- | --- | --- | --- |
|  | Young Adults (n=40) | Healthy Elderly (n=40) | Alzheimer’s Disease (n=20) | Semantic Dementia (n=3) | p | | Group Comparison |
| **Demographic** | | | | | | | |
| Age | 22.9 (3.3) | 73.85 (5.8) | 75.95 (6.4) | 66 (12.5) | p<0.001*** | | YA < HE = AD |
| Gender (F/M) | 28/12 | 28/12 | 14/6 | 1/2 | 1 | | YA = HE = AD |
| Education (in years) | 14.9 (2) | 13.2 (4.4) | 13.3 (4.2) | 13.33 (1.2) | 0.16 | | YA = HE = AD |
| **Global** | | | | | | | |
| MMSE | 29.9 (0.3) | 28.68 (1.2) | 22.65 (2.8) | 26.67 (2.1) | p<0.001*** | | YA > HE > AD |
| SRT (in ms) | 272.73 (26) | 302.56 (52.5) | 329.92 (35.1) | 296 (10.8) | p<0.001*** | | YA < HE = AD |
| **Memory (RL/RI-16** | | | | | | | |
| Immediate recall | 15.95 (0.2) | 15.3 (0.8) | 10.46 (3.8) |  | p<0.001*** | | YA = HE > AD |
| Sum free recall | 39.98 (4.1) | 29.48 (5.6) | 8.85 (8.3) |  | p<0.001*** | | YA > HE > AD |
| Sum total recall | 47.65 (0.6) | 46.3 (2.1) | 21.62 (14.8) |  | p<0.001*** | | YA = HE > AD |
| Delayed free recall | 15.45 (0.8) | 12.81 (2.2) | 3.25 (4) |  | p<0.001*** | | YA > HE > AD |
| Delayed total recall | 16 (0) | 15.85 (0.4) | 7.67 (4.9) |  | p<0.001*** | | YA = HE > AD |
| Recognition | 16 (0) | 15.89 (0.3) | 12.42 (3.3) |  | p<0.001*** | | YA = HE > AD |
| **Language** | | | | | | | |
| Fluency (Animal) | 28.05 (4.8) | 20.12 (4.1) | 12.05 (4.6) |  | p<0.001*** | | YA > HE > AD |
| Fluency (P) | 18.88 (4.4) | 15.15 (5.2) | 9.55 (4.6) |  | p<0.001*** | | YA > HE > AD |
| **Executive functions** | | | | | | | |
| TMT A (ms) | 23.15 (5.6) | 61.17 (27.7) | 102.78 (77.3) |  | | p<0.001*** | YA < HE < AD |
| TMT A (errors) | 0.02 (0.2) | 0.12 (0.3) | 1.05 (1.4) |  | | p<0.001*** | YA = HE > AD |
| TMT B (ms) | 47.6 (11.1) | 149.22 (70.4) | 230.43 (100.3) |  | | p<0.001*** | YA < HE < AD |
| TMT B (errors) | 0.35 (0.8) | 0.72 (1) | 2.71 (2.3) |  | | p<0.001*** | YA = HE > AD |
| Stroop (word) | 41.83 (6.1) | 52.38 (8.9) | 93.7 (59.6) |  | | p<0.001*** | YA = HE < AD |
| Stroop (color) | 60.4 (10.9) | 78.12 (18.1) | 78.2 (39.1) |  | | p<0.001*** | YA < HE = AD |
| Stroop (word-color) | 100.45 (18.8) | 147.7 (44) | 260.67 (126.1) |  | | p<0.001*** | YA < HE < AD |
| Stroop Errors  (word-color) | 0.18 (0.4) | 1.1 (2) | 6.05 (5.9) |  | | p<0.001*** | YA = HE > AD |
| *SD patients were excluded from the analyses due to the too limited sample size; p= p values computed for each ANOVA with the group (YA, HE, AD) as a between subject variable (or chi-squared test for the gender); *** = p < 0.001; MMSE= Mini-Mental State Examination; SRT=Simple Reaction Times; RL/RI= Rappel Libre/Rappel indicé 16 items; TMT= Trail Making Test; Group comparison= pairwise t-test to compare the YA, HE and AD groups one to each other; =, <, >= indicate how a given group performs compared to the following group(s).* | | | | | | | |
